# Prevalence and associated factors of vascular complications among inpatients with type 2 diabetes: A retrospective database study at a tertiary care department, Ningbo, China

**DOI:** 10.1371/journal.pone.0235161

**Published:** 2020-06-23

**Authors:** Jialin Li, Kaushik Chattopadhyay, Miao Xu, Yanshu Chen, Fangfang Hu, Jianping Chu, Li Li

**Affiliations:** 1 Department of Endocrinology and Metabolism, Ningbo First Hospital, Ningbo, People’s Republic of China; 2 Division of Epidemiology and Public Health, School of Medicine, University of Nottingham, Nottingham, United Kingdom; Suez Canal University Faculty of Medicine, EGYPT

## Abstract

To determine the prevalence of vascular complications among inpatients with type 2 diabetes mellitus (T2DM) and factors independently associated with vascular complications in a tertiary care department in Ningbo, China, the authors conducted a cross-sectional study using an existing computerised medical records database. A total of 3370 adult patients with T2DM were admitted to this tertiary care department for the first time between 2012 and 2017. Patients were categorised as those (1) with at least one vascular complication, (2) with at least one microvascular complication, and (3) with at least one macrovascular complication. Over 5 years, the prevalence of vascular, microvascular, and macrovascular complications among inpatients with T2DM was 73.2%, 57.5%, and 51.4%, respectively. The odds of vascular, microvascular, and macrovascular complications increased with age and were higher in patients with hypertension. The odds of vascular and microvascular complications were higher in single, divorced, or widowed patients, patients with T2DM for a long time, and patients on advanced T2DM therapeutic regimen. The odds of vascular and macrovascular complications were lower in women. The odds of microvascular complications decreased with education. The odds of macrovascular complications were higher in smokers. In conclusion, in the tertiary care department, more than half of inpatients with T2DM had vascular complications, and factors independently associated with vascular complications were identified. The study findings could be used in future interventional studies to prevent and manage vascular complications among these patients.

## Introduction

Type 2 diabetes mellitus (T2DM) is a chronic metabolic disorder. The health, social, and economic consequences of T2DM are enormous. Globally, China has the largest population with T2DM, with more than 116 million adults with T2DM, which will increase to approximately 147 million in 2045 [1]. Chronic hyperglycaemia in T2DM is related to microvascular complications (such as diabetic retinopathy, nephropathy, and neuropathy/foot), macrovascular complications (such as coronary heart disease [CHD], stroke, and peripheral arterial disease), poor quality of life, and even death [2,3]. In China, approximately one million deaths per year are attributed to T2DM and its associated complications. Nearly 40% of these are premature deaths (i.e. in adults <70 years of age) [4]. Currently, the healthcare expenditure due to T2DM is US $63 billion, and the total economic burden of T2DM and its complications is high [1,5,6].

Ningbo, located in the Zhejiang Province of China, is an economically developed city. With modernisation, the city has experienced an epidemiological transition from infectious diseases to chronic diseases [7]. In Ningbo, approximately 21% of people over the age of 40 years had T2DM in 2015 [8]. Many patients with T2DM who received treatment at the Department of Endocrinology and Metabolism, Ningbo First Hospital, had poor glycaemic control [9], which could lead to vascular complications.

To date, research on vascular complications among inpatients with T2DM has not been conducted at this tertiary care department. This study aimed to determine the prevalence of vascular complications among these patients and factors independently associated with vascular complications. Similar studies have been conducted in other populations and settings [10–17]. However, the evidence in this particular population and setting could be different from others, and this needs exploration and reporting. Even if the evidence is similar, it is important for policy and practice to replicate it. This context-specific information could be used by Chinese and/or international experts to develop, evaluate, and implement interventions for preventing and managing vascular complications locally and nationally. For example, UK- and China-based experts as part of the China Health Initiative, a unique cross-country collaboration between the UK and China, could use these findings to design future research studies. Similarly, many international funding agencies (such as the Medical Research Council, UK) funds global health research, and international experts could use these findings to design future research studies. Thus, even small robustly conducted studies should be disseminated to make the evidence-base strong.

## Materials and methods

### Study setting, design, data source, and period

The study was conducted at the Department of Endocrinology and Metabolism, Ningbo First Hospital, China. The main responsibilities of this tertiary care hospital are to provide specialist health and medical services and perform a larger role in health and medical research and education [18,19]. The authors conducted a cross-sectional study using an existing computerised medical records database that was developed for this tertiary care department by the Yinal Software Corporation, China. As this is a medical records database, the medico-nursing team is responsible for data entry on to the database. Another independent team of hospital staff checks data quality and is responsible for the overall management of the database. The study period extended from 1 July 2012 to 30 June 2017 (5 years) and included 6699 patients in the database.

### Study population and inclusion and exclusion criteria

In China, local people and people from surrounding areas can visit any hospital of their choice without a referral from a primary care physician [18]. The study included adult patients (≥18 years of age) with T2DM who were admitted to the tertiary care department for the first time. The management of uncontrolled blood glucose levels was the main reason for hospitalisation of patients with T2DM. They were also tested for any new or existing vascular complication (i.e. three microvascular complications and three macrovascular complications). These complications were diagnosed by a team of qualified and experienced experts at the tertiary care department, using national standard guidelines [20–23]. The guidelines have been developed keeping in mind the national healthcare setting and the native Chinese population. Diabetic retinopathy was diagnosed by ophthalmologists based on funduscopy or fundus fluorescence findings [20]. An albumin-to-creatinine ratio ≥2.5 mg/mmol in men/≥3.5 mg/mmol in women or an estimated glomerular filtration rate <60 ml/min per 1.73 m^2^ confirmed the diagnosis of diabetic nephropathy [20]. The diagnosis of diabetic neuropathy was based on typical symptoms and physical examination or nerve conduction function examination results if the patient had no signs or symptoms [20]. The diagnosis of a diabetic foot was based on signs and symptoms (clinical findings) and having diabetic peripheral neuropathy or peripheral arterial stenosis [20,21]. CHD included angina or myocardial infarction with the diagnosis based on signs and symptoms and further confirmed through coronary angiography or 320-slice spiral computed tomography [22]. The diagnosis of stroke (ischaemic stroke) was based on signs and symptoms and further confirmed through magnetic resonance imaging [23]. Those with peripheral arterial disease had dull or cramping leg pain and had a plaque in the peripheral vessel, vascular stenosis, or occlusion, as confirmed by ultrasonography [20]. Patients diagnosed with gestational diabetes, type 1 diabetes, secondary diabetes, unknown type of diabetes, or endocrine disease (e.g. Cushing syndrome and hyperthyroidism) were excluded. The study inclusion criteria were satisfied by 3370 patients.

### Study variables

The following independent variables (measured at the time of the first admission to the tertiary care department) were extracted from the database: age, sex, education, occupation, marital status, residence (based on the “hukou” system i.e. residence registration system in China) [24], health insurance, smoking (current status), alcohol drinking (current status), family history of T2DM (any parent or sibling), T2DM duration, blood glucose level (glycated haemoglobin [HbA1c]; estimated using high-performance liquid chromatography [D-10 Hemoglobin Analyzer, Bio-Rad, USA]), T2DM therapeutic regimen [20], body mass index (BMI) [25], hypertension (diagnosis based on blood pressure measurement ≥140/90 mm Hg or self-reported history of hypertension and on antihypertensive therapy) [26], and hyperlipidaemia (diagnosis based on serum lipids: total cholesterol ≥4.5 mmol/L or triglycerides ≥1.7 mmol/L) [20]. The dependent variables extracted were vascular complications of T2DM. Patients were categorised as those (1) with at least one vascular complication, (2) with at least one microvascular complication, and (3) with at least one macrovascular complication. Data on sodium-glucose co-transporter 2 inhibitors, dipeptidyl peptidase 4 inhibitors, and glucagon-like peptide 1 receptor agonists were unavailable in the database. During the study period, these drugs were not sold in this hospital because they were not approved by the China Food and Drug Administration or not covered by the Chinese health insurance system [27].

### Ethics

The Research Ethics Committee of the Ningbo First Hospital, China, approved this study. The authors had no access to information that could identify individual participants during data analyses. According to the research ethics rules, no informed consent was necessary.

### Statistical analyses

Numbers and percentages were calculated for categorical variables. For a normally distributed continuous variable, the mean ± standard deviation was calculated. To find any independent association between independent variables and vascular complications, multiple logistic regression models were developed and independent variables with p ≤ 0.20 in simple logistic regressions (see S1 File) were included. The authors calculated odds ratios (ORs) and 95% confidence intervals (CIs). The authors used IBM SPSS Statistics version 20.0 for Windows for data analyses.

## Results

Table 1 reports the characteristics of inpatients with T2DM with and without vascular complications. The mean ± standard deviation age of patients with T2DM was 62.9 ± 13.8 years, and nearly 51% (n = 1713) were men. Over a period of 5 years, the prevalence of vascular, microvascular, and macrovascular complications among patients with T2DM was 73.2% (n = 2466), 57.5% (n = 1939), and 51.4% (n = 1731), respectively. In total, 25.3% (n = 851), 29.7% (n = 1000), and 33.1% (n = 1115) had diabetic retinopathy, nephropathy, and neuropathy/foot, respectively. Similarly, 7.7% (n = 259), 10.1% (n = 342), and 44.1% (n = 1487) had CHD, stroke, and peripheral arterial disease, respectively.

**Table 1:** Characteristics of inpatients with T2DM with and without vascular complications.

	Vascular complications		Microvascular complications		Macrovascular Complications	
	No, 904 (26.8) *	Yes, 2466 (73.2) *	No, 1431 (42.5) *	Yes, 1939 (57.5) *	No, 1639 (48.6) *	Yes, 1731 (51.4) *
**Age (years)**						
18–39	173 (84.4)	32 (15.6)	177 (86.3)	28 (13.7)	195 (95.1)	10 (4.9)
40–59	409 (39.2)	634 (60.8)	559 (53.6)	484 (46.4)	673 (64.5)	370 (35.5)
≥60	322 (15.2)	1800 (84.8)	695 (32.8)	1427 (67.2)	771 (36.3)	1315 (63.7)
**Sex**						
Male	442 (25.8)	1271 (74.2)	736 (43.0)	977 (57.0)	786 (45.9)	927 (54.1)
Female	462 (27.9)	1195 (72.1)	695 (41.9)	962 (58.1)	853 (51.5)	804 (48.5)
**Education**						
University/college	148 (42.0)	204 (58.0)	217 (61.6)	135 (38.4)	201 (57.1)	151 (42.9)
Class 7–12	396 (30.0)	926 (70.0)	612 (46.3)	710 (53.7)	677 (51.2)	645 (48.8)
Class 1–6	254 (22.2)	890 (77.8)	426 (37.2)	718 (62.8)	510 (44.6)	634 (55.4)
No qualification	106 (19.2)	446 (80.8)	176 (31.9)	376 (68.1)	251 (45.5)	301 (54.5)
**Occupation**						
Never worked/retired	356 (19.0)	1516 (81.0)	654 (34.9)	1218 (65.1)	780 (41.7)	1092 (58.3)
Non-manual worker	343 (44.5)	428 (55.5)	460 (59.7)	311 (40.3)	494 (64.1)	277 (35.9)
Manual worker	205 (28.2)	522 (71.8)	317 (43.6)	410 (56.4)	365 (50.2)	362 (49.8)
**Marital status**						
Married	799 (27.6)	2096 (72.4)	1267 (43.8)	1628 (56.2)	1412 (48.8)	1483 (51.2)
Single/divorced/widowed	105 (22.1)	370 (77.9)	164 (34.5)	311 (65.5)	227 (47.8)	248 (52.2)
**Residence**						
Urban	500 (25.2)	1488 (74.8)	837 (42.1)	1151 (57.9)	914 (46.0)	1074 (54.0)
Rural	404 (29.2)	978 (70.8)	594 (43.0)	788 (57.0)	725 (52.5)	657 (47.5)
**Health insurance**						
Yes	727 (25.2)	2155 (74.8)	1190 (41.3)	1692 (58.7)	1350 (46.8)	1532 (53.2)
No	177 (36.3)	311 (63.7)	241 (49.4)	247 (50.6)	289 (59.2)	199 (40.8)
**Smoking (current status)**						
No	735 (27.1)	1978 (72.9)	1132 (41.7)	1581 (58.3)	1353 (49.9)	1360 (50.1)
Yes	169 (25.7)	488 (74.3)	299 (45.5)	358 (54.5)	286 (43.5)	371 (56.5)
**Alcohol drinking (current status)**						
No	826 (27.5)	2179 (72.5)	1286 (42.8)	1719 (57.2)	1487 (49.5)	1518 (50.5)
Yes	78 (21.4)	287 (78.6)	145 (39.7)	220 (60.3)	152 (41.6)	213 (58.4)
**Family history of T2DM (any parent or sibling)**						
No	578 (26.3)	1616 (73.7)	927 (42.3)	1267 (57.7)	1059 (48.3)	1135 (51.7)
Yes	326 (27.7)	850 (72.3)	504 (42.9)	672 (57.1)	580 (49.3)	596 (50.7)
**T2DM duration (years)**						
≤1	69 (49.3)	71 (50.7)	100 (71.4)	40 (28.6)	87 (62.1)	53 (37.9)
>1–5	365 (46.8)	415 (53.2)	502 (64.4)	278 (35.6)	514 (65.9)	266 (34.1)
>5–10	226 (30.3)	520 (69.7)	339 (45.4)	407 (54.6)	390 (52.3)	356 (47.7)
>10	244 (14.3)	1460 (85.7)	490 (28.8)	1214 (71.2)	648 (38.0)	1056 (62.0)
**Blood glucose level (HbA1c, %)**						
<7%	171 (28.4)	432 (71.6)	271 (44.9)	332 (55.1)	300 (49.8)	303 (50.2)
≥7%	699 (26.1)	1979 (73.9)	1112 (41.5)	1566 (58.5)	1287 (48.1)	1391 (51.9)
Unknown	34 (38.2)	55 (61.8)	48 (53.9)	41 (46.1)	52 (58.4)	37 (41.6)
**T2DM therapeutic regimen**						
Lifestyle modification^i^ only	149 (40.5)	219 (59.5)	207 (56.2)	161 (43.8)	211 (57.3)	157 (42.7)
Lifestyle modification^i^ + OAD^ii^	250 (30.0)	584 (70.0)	401 (48.1)	433 (51.9)	412 (49.4)	422 (50.6)
Lifestyle modification^i^ + insulin^iii^	111 (27.6)	291 (72.4)	172 (42.8)	230 (57.2)	205 (51.0)	197 (49.0)
Lifestyle modification^i^ + OAD^ii^ + insulin^iii^	394 (22.3)	1372 (77.7)	651 (36.9)	1115 (63.1)	811 (45.9)	955 (54.1)
**BMI (kg/m**^**2**^**)**						
18.5–23.9	363 (25.0)	1087 (75.0)	589 (40.6)	861 (59.4)	687 (47.4)	763 (52.6)
<18.5	25 (18.7)	109 (81.3)	43 (32.1)	91 (67.9)	67 (50.0)	67 (50.0)
≥24.0	492 (29.8)	1158 (70.2)	753 (45.6)	897 (54.4)	835 (50.6)	815 (49.4)
Unknown	24 (17.6)	112 (82.4)	46 (33.8)	90 (66.2)	50 (36.8)	86 (63.2)
**Hypertension**						
No	398 (39.7)	605 (60.3)	537 (53.5)	466 (46.5)	619 (61.7)	384 (38.3)
Yes	506 (21.4)	1861 (78.6)	894 (37.8)	1473 (62.2)	1020 (43.1)	1347 (56.9)
**Hyperlipidaemia**						
No	184 (22.3)	641 (77.7)	326 (39.5)	499 (60.5)	384 (46.5)	441 (53.5)
Yes	706 (28.0)	1814 (72.0)	1083 (43.0)	1437 (57.0)	1238 (49.1)	1282 (50.9)
Unknown	14 (56.0)	11 (44.0)	22 (88.0)	3 (12.0)	17 (68.0)	8 (32.0)

BMI, body mass index; HbA1c, glycated haemoglobin; OAD, oral antidiabetic drug; T2DM, type 2 diabetes mellitus.

^i^diet and physical activity.

^ii^metformin, acarbose, sulfonylureas, meglitinides, and/or thiazolidinediones.

^iii^long-term insulin, intermediate insulin, rapid-acting insulin, and/or premix insulin.

*n (%).

Table 2 reports the multiple logistic regression analyses—independent variables with p ≤ 0.20 in simple logistic regressions were included. The data below are reported as OR, 95% CI. The odds of vascular complications increased with age (18–39 years: 1; 40–59 years: 6.54, 4.31–9.92; and ≥60 years: 15.98, 10.30–24.81); were higher in single, divorced, or widowed patients than in married patients (1.48, 1.12–1.96), patients having T2DM for >10 years compared to ≤1 year (2.75, 1.81–4.17), patients on lifestyle modification + oral antidiabetic drug (OAD) + insulin compared to lifestyle modification alone (1.54, 1.15–2.06), and patients with hypertension (1.50, 1.25–1.81); and were lower in women than in men (0.65, 0.54–0.79).

**Table 2:** Multiple logistic regression analyses—independent variables with p ≤ 0.20 in simple logistic regression were included.

	Adjusted OR (95% CI)
**Vascular complications**^a^	
**Age (years)**	
18–39	1
40–59	6.54 (4.31, 9.92)
≥60	15.98 (10.30, 24.81)
**Sex**	
Male	1
Female	0.65 (0.54, 0.79)
**Occupation**	
Never worked/retired	1
Non-manual worker	0.81 (0.63, 1.04)
Manual worker	1.06 (0.83, 1.34)
**Marital status**	
Married	1
Single/divorced/widowed	1.48 (1.12, 1.96)
**T2DM duration (years)**	
≤1	1
>1–5	0.91 (0.60, 1.37)
>5–10	1.47 (0.96, 2.23)
>10	2.75 (1.81, 4.17)
**Blood glucose level (HbA1c, %)**	
<7%	1
≥7%	1.13 (0.89, 1.43)
Unknown	0.61 (0.36, 1.02)
**T2DM therapeutic regimen**	
Lifestyle modification^i^ only	1
Lifestyle modification^i^ + OAD^ii^	1.20 (0.89, 1.63)
Lifestyle modification^i^ + insulin^iii^	1.34 (0.94, 1.91)
Lifestyle modification^i^ + OAD^ii^ + insulin^iii^	1.54 (1.15, 2.06)
**Hypertension**	
No	1
Yes	1.50 (1.25, 1.81)
**Hyperlipidaemia**	
No	1
Yes	0.91 (0.74, 1.13)
Unknown	0.28 (0.11, 0.69)
**Microvascular complications**^b^	
**Age (years)**	
18–39	1
40–59	3.50 (2.26, 5.42)
≥60	5.04 (3.20, 7.96)
**Education**	
University/college	1
Class 7–12	1.46 (1.11, 1.92)
Class 1–6	1.63 (1.22, 2.17)
No qualification	1.95 (1.41, 2.71)
**Occupation**	
Never worked/retired	1
Non-manual worker	0.81 (0.64, 1.02)
Manual worker	1.07 (0.87, 1.30)
**Marital status**	
Married	1
Single/divorced/widowed	1.41 (1.12, 1.77)
**T2DM duration (years)**	
≤1	1
>1–5	1.16 (0.76, 1.76)
>5–10	2.22 (1.46, 3.38)
>10	3.70 (2.45, 5.57)
**Blood glucose level (HbA1c, %)**	
<7%	1
≥7%	1.11 (0.90, 1.36)
Unknown	0.67 (0.41, 1.08)
**T2DM therapeutic regimen**	
Lifestyle modification^i^ only	1
Lifestyle modification^i^ + OAD^ii^	1.15 (0.87, 1.51)
Lifestyle modification^i^ + insulin^iii^	1.42 (1.03, 1.95)
Lifestyle modification^i^ + OAD^ii^ + insulin^iii^	1.57 (1.21, 2.03)
**Hypertension**	
No	1
Yes	1.28 (1.09, 1.52)
**Hyperlipidaemia**	
No	1
Yes	0.98 (0.82, 1.17)
Unknown	0.09 (0.03, 0.32)
**Macrovascular complications**^c^	
**Age (years)**	
18–39	1
40–59	8.47 (4.40, 16.29)
≥60	24.14 (12.57, 46.34)
**Sex**	
Male	1
Female	0.71 (0.60, 0.84)
**Smoking (current status)**	
No	1
Yes	1.37 (1.11, 1.70)
**T2DM duration (in years)**	
≤1	1
>1–5	0.76 (0.50, 1.15)
>5–10	1.07 (0.71, 1.61)
>10	1.47 (0.99, 2.20)
**Blood glucose level (HbA1c, %)**	
<7%	1
≥7%	1.14 (0.94, 1.38)
Unknown	0.71 (0.44, 1.15)
**Hypertension**	
No	1
Yes	1.47 (1.24, 1.74)

BMI, body mass index; CI, confidence interval; HbA1c, glycated haemoglobin; OAD, oral antidiabetic drug; OR, odds ratio; T2DM, type 2 diabetes mellitus.

^a^Variables included age, sex, education, occupation, marital status, residence, health insurance, alcohol drinking, T2DM duration, blood glucose level (HbA1c), T2DM therapeutic regimen, BMI, hypertension, and hyperlipidaemia.

^b^Variables included age, education, occupation, marital status, health insurance, smoking, T2DM duration, blood glucose level (HbA1c), T2DM therapeutic regimen, BMI, hypertension, and hyperlipidaemia.

^c^Variables included age, sex, education, occupation, residence, health insurance, smoking, alcohol drinking, T2DM duration, blood glucose level (HbA1c), T2DM therapeutic regimen, BMI, hypertension, and hyperlipidaemia.

^i^diet and physical activity.

^ii^metformin, acarbose, sulfonylureas, meglitinides, and/or thiazolidinediones.

^iii^long-term insulin, intermediate insulin, rapid-acting insulin, and/or premix insulin.

The odds of microvascular complications increased with age (18–39 years: 1; 40–59 years: 3.50, 2.26–5.42; and ≥60 years: 5.04, 3.20–7.96); decreased with education (university/college: 1; class 7–12: 1.46, 1.11–1.92; class 1–6: 1.63, 1.22–2.17; and no qualification: 1.95, 1.41–2.71); and were higher in single, divorced, or widowed patients than in married patients (1.41, 1.12–1.77), patients having T2DM for >5–10 years and >10 years compared to ≤1 year (2.22, 1.46–3.38; and 3.70, 2.45–5.57, respectively), patients on lifestyle modification + insulin and lifestyle modification + OAD + insulin compared to lifestyle modification alone (1.42, 1.03–1.95; and1.57, 1.21–2.03, respectively), and patients with hypertension (1.28, 1.09–1.52).

The odds of macrovascular complications increased with age (18–39 years: 1; 40–59 years: 8.47, 4.40–16.29; and ≥60 years: 24.14, 12.57–46.34); were higher in smokers (1.37, 1.11–1.70) and patients with hypertension (1.47, 1.24–1.74); and were lower in women than in men (0.71, 0.60–0.84).

## Discussion

In the tertiary care department, more than half of inpatients with T2DM had vascular complications, indicating the need for interventions to prevent and manage vascular complications in this setting and the population. Major inpatient-based studies conducted in China have reported a relatively higher prevalence of vascular complications [10,11]. On the other hand, major outpatient-based studies conducted in China have reported a comparatively lower prevalence of vascular complications [12,13]. For example, in 2007, a survey conducted in four major Chinese cities showed the prevalence of microvascular and macrovascular complications was 34.7% and 33.4%, respectively [12]. The probable reasons behind such variations are different population characteristics (e.g. disease severity will determine hospital admission), different study periods, and different case definitions used for vascular complications. Similar studies have been conducted worldwide in a range of settings and populations. The prevalence figures are consistent with some studies and are not consistent with others [16,28]. In terms of ethnicity and geographical location, multi-country studies found a difference in the pattern of vascular complications [29–31]. For example, a higher prevalence of microvascular complications and a lower prevalence of macrovascular complications were found among Chinese people compared to Europeans [29,30]. Thus, a systematic review should be conducted to summarise the prevalence and associated factors of vascular complications in different geographical locations.

This study findings that the odds of vascular, microvascular, and macrovascular complications increased with age and were lower in women (except in the case of microvascular complications) are consistent with previous studies [28,32–36]. It has also been reported that older and younger patients are at risk of developing a similar spectrum of vascular complications. Men are usually at a higher risk of microvascular complications, whereas the consequences of macrovascular complications might be greater in women. Interestingly, in the absence of T2DM, women have a far lower risk of vascular complications for much of their lifespan. However, the presence of T2DM confers a greater risk for vascular complications in women, and some of the potential reasons include the contribution of sex hormones and sex-specific risk factors [37]. Nevertheless, in this study setting and population, older men need more attention in terms of preventing and managing their vascular complications.

The finding that the odds of microvascular complications decreased with education is consistent with previous research [38–40]. Higher education levels usually help in patient education and behavioural changes [41]. The beneficial effect of education could be visible throughout the T2DM management pathway, including access to healthcare and uptake and adherence to health/medical advice and therapies [13,42]. The finding that the odds of vascular and microvascular complications were higher in single, divorced, or widowed patients is also consistent with previous research [43]. Married people usually have their spouse to look after them, both physically and mentally, which in turn improves their access to healthcare and uptake and adherence to health/medical advice and therapies [44,45]. In this study, the odds of macrovascular complications were higher in smokers. Smoking is a well-researched risk factor for CHD, stroke, and peripheral arterial disease, both in the general population and in patients with T2DM [46,47]. Smoking cessation, as part of a healthy lifestyle, plays a major role in reducing the risk of these diseases [46,48].

In this study, the odds of vascular and microvascular complications were higher in patients with T2DM for >10 and >5 years, respectively. The risk of vascular complications increases with T2DM duration [12,28,33,35,38]. The mass and function of beta-cells gradually decline with the progression of T2DM, leading to vascular complications [49]. It should be noted that undiagnosed T2DM is a major problem, and the exact duration of T2DM is often unknown [50]. In a considerable number of patients, vascular complications are detected at the same time as the diagnosis of T2DM [51,52]. The finding that the odds of vascular and microvascular complications were higher in patients on lifestyle modification + OAD + insulin as part of their T2DM therapeutic regimen is consistent with previous research [53]. In addition, in this study, the odds of microvascular complications were higher in patients on lifestyle modification + insulin. A stepwise approach is recommended in the Chinese T2DM management guideline [20]. The first step is lifestyle modification. With disease progression, OAD(s) and/or insulin(s) are added. Therefore, the identified association is more likely to represent disease severity and chronicity than medication effects. It should be noted that the uptake and adherence to the T2DM therapeutic regimen could be different from what is prescribed [54,55]. Recently, approximately 43% of patients with T2DM in China were found to comply with their T2DM therapeutic regimen [56]. In this study database, prescription data were available, but uptake and adherence data were unavailable, which needs to be explored and considered in future studies. In this study, the odds of vascular, microvascular, and macrovascular complications were higher in patients with hypertension. Hypertension is a well-researched risk factor for vascular complications [28,35,38,57]. In the UK Prospective Diabetes Study, each 10 mm Hg decrease in updated mean systolic blood pressure was associated with a reduction in risk of 12% for any vascular complication, 13% for microvascular complications, and 11% for myocardial infarction [58]. Thus, the importance of antihypertensive therapy in patients with hypertension cannot be ignored.

Although both microvascular and macrovascular complications are vascular diseases and share some common risk factors, the pathophysiology and clinical manifestations differ [59]. Some of the risk factors are unique to each of these two. In this study, middle-aged and older patients and those with hypertension had higher microvascular and macrovascular complications. In addition, those who had a lower education level, were single, divorced, or widowed, had T2DM for a long time and were on an advanced T2DM therapeutic regimen had higher microvascular complications. Similarly, men and smokers had higher macrovascular complications.

Hyperglycaemia and hyperlipidaemia are known risk factors for vascular complications [10,11,14]. However, these associations were not significant in this study. Vascular complications develop over a period of time [12,28,33,35,38]. However, in this study, data on blood glucose and serum lipids came from a single point in time and did not reflect their long-term patterns. Postprandial blood glucose levels might be a better predictor of T2DM complications, especially macrovascular complications [60,61]. However, data on postprandial blood glucose were of poor quality.

This study was the first to explore vascular complications among inpatients with T2DM in a tertiary care department in Ningbo, China. As far as the authors are aware, it was the first time an existing computerised medical records database was used for this purpose in the Zhejiang Province of China. Microvascular and macrovascular complications of T2DM were explored, which in turn provided a more complete picture. The study findings could be valid in similar settings and populations. The authors conducted a hospital-based study, and a different picture could emerge from a population-based study because of differences in setting and population. Missing data were generally low in this study. Samples with missing values for the variables were included in the logistic regression analyses. This retrospective study used an existing computerised medical records database; its main purpose is clinical and not research. Therefore, data quality issues of routinely collected data could be present. In general, inpatients are precisely monitored owing to disease severity, and this could have improved the data quality. The diagnosis of CHD, stroke, and peripheral arterial disease was based on signs and symptoms, followed by diagnostic tests. Globally, this is what is usually done in clinical practice, and the authors might have missed subclinical patients. The study findings could be due to other factors missing in the database (e.g. uptake and adherence to the T2DM therapeutic regimen [62]); these factors were not adjusted for in the multiple logistic regression analyses. Recall bias could be an issue in self-reported data (e.g. family history of T2DM). This erroneous variable measurement could have assigned people to the wrong category, resulting in an incorrect assessment of the relationship between family history of T2DM and vascular complications. In this cross-sectional study, it was impossible to determine the causal association between variables and vascular complications. Thus, a long-term, longitudinal study should be conducted to explore the effects of these and other potential factors on vascular complications.

## Conclusions

In the tertiary care department in Ningbo, China, more than half of inpatients with T2DM had vascular complications, and factors independently associated with vascular complications were identified. Vascular, microvascular, and macrovascular complications increased with age and were higher in patients with hypertension. In single, divorced, or widowed patients, patients with T2DM for a long time, and patients on advanced T2DM therapeutic regimen, vascular and microvascular complications were found to be higher. In women, vascular and macrovascular complications were found to be lower. Microvascular complications decreased with education. In smokers, macrovascular complications were found to be higher.

## Implications for policy and clinical practice

The context-specific information could be used to develop, evaluate, and implement interventions for preventing and managing vascular complications. Overall, in clinical practice, vascular complication prevention and management strategies in the given setting and the population need to be targeted towards middle-aged and older adults, men, smokers, those who have a lower education level, have never worked or are retired, are single, divorced, or widowed, have comorbidities such as hypertension, have T2DM for a long time, or are on an advanced T2DM therapeutic regimen.

## Supporting information

S1 File Simple logistic regression analyses. (DOCX)

S1 Dataset Vascular complications among inpatients with type 2 diabetes in Ningbo, China. (XLSX)
