# Supplementary material for: Prevalence and associated factors of vascular complications among inpatients with type 2 diabetes: A retrospective database study at a tertiary care department, Ningbo, China
Source: PLoS One. 2020 Jun 23;15(6):e0235161. doi: 10.1371/journal.pone.0235161 (PMC7310722; doi:10.1371/journal.pone.0235161)
Supplement: S1 File — (DOCX) [file pone.0235161.s001.docx]

S1. Simple logistic regression analyses.

|  | **Vascular**  **Complications** | | | **Microvascular**  **complications** | | **Macrovascular**  **Complications** | |
| --- | --- | --- | --- | --- | --- | --- | --- |
|  | **Unadjusted OR (95% CI)** | | **p value** | **Unadjusted OR (95% CI)** | **p value** | **Unadjusted OR (95% CI)** | **p value** |
| **Age (years)** |  | <0.001 | |  | <0.001 |  | <0.001 |
| 18-39 | 1 |  | | 1 |  | 1 |  |
| 40-59 | 8.38 (5.63, 12.45) |  | | 5.47 (3.61, 8.30) |  | 10.72 (5.61, 20.50) |  |
| ≥60 | 30.22 (20.35, 44.88) |  | | 12.98 (8.62, 19.53) |  | 34.17 (17.99, 64.91) |  |
| **Sex** |  | 0.173 | |  | 0.548 |  | 0.001 |
| Male | 1 |  | | 1 |  | 1 |  |
| Female | 0.90 (0.77, 1.05) |  | | 1.04 (0.91, 1.20) |  | 0.80 (0.70, 0.92) |  |
| **Education** |  | <0.001 | |  | <0.001 |  | <0.001 |
| University/college | 1 |  | | 1 |  | 1 |  |
| Class 7-12 | 1.70 (1.33, 2.16) |  | | 1.87 (1.47, 2.37) |  | 1.27 (1.01, 1.61) |  |
| Class 1-6 | 2.54 (1.97, 3.28) |  | | 2.71 (2.12, 3.47) |  | 1.66 (1.30, 2.11) |  |
| No qualification | 3.05 (2.26, 4.12) |  | | 3.43 (2.60, 4.54) |  | 1.60 (1.22, 2.09) |  |
| **Occupation** |  | <0.001 | |  | <0.001 |  | <0.001 |
| Never worked/retired | 1 |  | | 1 |  | 1 |  |
| Non-manual worker | 0.29 (0.24, 0.35) |  | | 0.36 (0.31, 0.43) |  | 0.40 (0.34, 0.48) |  |
| Manual worker | 0.60 (0.49, 0.73) |  | | 0.69 (0.58, 0.83) |  | 0.71 (0.60, 0.84) |  |
| **Marital status** |  | 0.012 | |  | <0.001 |  | 0.691 |
| Married | 1 |  | | 1 |  | 1 |  |
| Single/divorced/widowed | 1.34 (1.07, 1.69) |  | | 1.48 (1.21, 1.81) |  | 1.04 (0.86, 1.26) |  |
| **Residence** |  | 0.009 | |  | 0.612 |  | <0.001 |
| Urban | 1 |  | | 1 |  | 1 |  |
| Rural | 0.81 (0.70, 0.95) |  | | 0.97 (0.84, 1.11) |  | 0.77 (0.67, 0.89) |  |
| **Health insurance** |  | <0.001 | |  | 0.001 |  | <0.001 |
| Yes | 1 |  | | 1 |  | 1 |  |
| No | 0.59 (0.48, 0.73) |  | | 0.72 (0.60, 0.87) |  | 0.61 (0.50, 0.74) |  |
| **Smoking (current status)** |  | 0.477 | |  | 0.078 |  | 0.004 |
| No | 1 |  | | 1 |  | 1 |  |
| Yes | 1.07 (0.88, 1.30) |  | | 0.86 (0.72, 1.02) |  | 1.29 (1.09, 1.53) |  |
| **Alcohol drinking (current status)** |  | 0.013 | |  | 0.263 |  | 0.005 |
| No | 1 |  | | 1 |  | 1 |  |
| Yes | 1.40 (1.07, 1.81) |  | | 1.14 (0.91, 1.42) |  | 1.37 (1.10, 1.71) |  |
| **Family history of T2DM (any parent or sibling)** |  | 0.390 | |  | 0.735 |  | 0.560 |
| No | 1 |  | | 1 |  | 1 |  |
| Yes | 0.93 (0.80, 1.09) |  | | 0.98 (0.85, 1.13) |  | 0.96 (0.83, 1.11) |  |
| **T2DM duration (years)** |  | <0.001 | |  | <0.001 |  | <0.001 |
| ≤1 | 1 |  | | 1 |  | 1 |  |
| >1-5 | 1.11 (0.77, 1.58) |  | | 1.38 (0.93, 2.06) |  | 0.85 (0.59, 1.23) |  |
| >5-10 | 2.24 (1.55, 3.23) |  | | 3.00 (2.02, 4.45) |  | 1.50 (1.03, 2.17) |  |
| >10 | 5.82 (4.07, 8.32) |  | | 6.19 (4.23, 9.07) |  | 2.68 (1.88, 3.82) |  |
| **Blood glucose level (HbA1c, %)** |  | 0.028 | |  | 0.027 |  | 0.133 |
| <7% | 1 |  | | 1 |  | 1 |  |
| ≥7% | 1.12 (0.92, 1.37) |  | | 1.15 (0.96, 1.37) |  | 1.07 (0.90, 1.28) |  |
| Unknown | 0.64 (0.40, 1.02) |  | | 0.70 (0.45, 1.09) |  | 0.70 (0.45, 1.11) |  |
| **T2DM therapeutic regimen** |  | <0.001 | |  | <0.001 |  | 0.001 |
| Lifestyle modification^i^ only | 1 |  | | 1 |  | 1 |  |
| Lifestyle modification^i^ + OAD^ii^ | 1.59 (1.23, 2.05) |  | | 1.39 (1.09, 1.78) |  | 1.38 (1.08, 1.76) |  |
| Lifestyle modification^i^ + insulin^iii^ | 1.78 (1.32, 2.41) |  | | 1.72 (1.29, 2.29) |  | 1.29 (0.97, 1.72) |  |
| Lifestyle modification^i^ + OAD^ii^ + insulin^iii^ | 2.37 (1.87, 3.00) |  | | 2.20 (1.75, 2.77) |  | 1.58 (1.26, 1.99) |  |
| **BMI (kg/m^2^)** |  | <0.001 | |  | <0.001 |  | 0.011 |
| 18.5-23.9 | 1 |  | | 1 |  | 1 |  |
| <18.5 | 1.46 (0.93, 2.29) |  | | 1.45 (0.99, 2.11) |  | 0.90 (0.63, 1.28) |  |
| ≥24.0 | 0.79 (0.67, 0.92) |  | | 0.82 (0.71, 0.94) |  | 0.88 (0.76, 1.01) |  |
| Unknown | 1.56 (0.99, 2.46) |  | | 1.34 (0.92, 1.94) |  | 1.55 (1.08, 2.23) |  |
| **Hypertension** |  | <0.001 | |  | <0.001 |  | <0.001 |
| No | 1 |  | | 1 |  | 1 |  |
| Yes | 2.42 (2.06, 2.84) |  | | 1.90 (1.64, 2.21) |  | 2.13 (1.83, 2.48) |  |
| **Hyperlipidaemia** |  | <0.001 | |  | <0.001 |  | 0.086 |
| No | 1 |  | | 1 |  | 1 |  |
| Yes | 0.74 (0.61, 0.89) |  | | 0.87 (0.74, 1.02) |  | 0.90 (0.77, 1.06) |  |
| Unknown | 0.23 (0.10, 0.51) |  | | 0.09 (0.03, 0.30) |  | 0.41 (0.18, 0.96) |  |

BMI, body mass index; HbA1c, glycated haemoglobin; OAD, oral antidiabetic drug; T2DM, type 2 diabetes mellitus.

^i^diet and physical activity.

^ii^metformin, acarbose, sulfonylureas, meglitinides, and/or thiazolidinediones.

^iii^long-term insulin, intermediate insulin, rapid-acting insulin, and/or premix insulin.
